# CD45 on CD33br HLA-DR+ immune cells promote cholecystitis via glycine-mediated pathways: A Mendelian randomization study

**DOI:** 10.1097/MD.0000000000044248

**Published:** 2025-09-19

**Authors:** Xiangrui Zeng, Rui Song, Zheng Zhang, Jinni Yao, Heqiang Liao, Congyu Wang, Zhe Xu, Huaicheng Yang

**Affiliations:** a Graduate School of Bengbu Medical University, Bengbu, China; b Department of Hepatobiliary and Pancreatic Surgery, First Affiliated Hospital of Anhui University of Science and Technology, Huainan, China; c First Clinical College of Anhui University of Science and Technology, Huainan, China.

**Keywords:** CD45 on CD33br HLA-DR+, cholecystitis, glycine levels, Mendelian randomization

## Abstract

Over the past 2 decades, the prevalence of cholecystitis has increased significantly, a trend believed to be influenced by improvements in quality of life and changes in lifestyle habits. However, the precise etiology of cholecystitis remains unclear. Although gallstones are commonly associated with this condition, recent studies suggest that immune cells also play a critical role in their development. This study investigated the relationship between cholecystitis and CD45 on CD33br HLA-DR+ immune cells, focusing on the mediating role of glycine levels, using Mendelian randomization (MR). The primary analytical approach employed was inverse variance weighting (IVW) complemented by additional methods, such as MR-Egger, weighted median, simple mode, and weighted mode. Horizontal pleiotropy and heterogeneity were evaluated to ensure the robustness of the MR findings, and a “leave-one-out” analysis was conducted to explore potential mediating effects. IVW analysis revealed that the association between cholecystitis and CD45 on CD33br HLA-DR+ immune cells yielded an odds ratio of 1.039 (95% confidence interval: 1.012–1.067, *P* = .004). Mediation analysis further indicated that glycine levels mediated this relationship, with a significant mediating effect of 6.87% (*P* = .0046). Notably, cholecystitis did not exhibit a reverse causal effect of CD45 on CD33br HLA-DR+ levels (*P* = .545; IVW odds ratio = 1.044, 95% confidence interval: 0.907–1.201). These findings suggested that the association between CD45 on CD33br HLA-DR+ immune cells and cholecystitis is mediated by glycine levels. This study provides novel insights into cholecystitis pathogenesis, highlighting the potential role of metabolic factors in immune-mediated inflammation. These results offer a new perspective on the underlying mechanisms of cholecystitis and suggest potential metabolic intervention targets for its prevention.

## 1. Introduction

Cholecystitis is a common digestive disorder that significantly affects the quality of life and exhibits an age-related increase in prevalence. Recent studies have highlighted concerns about the rise in the incidence of cholecystitis among children.^[1]^ In recent decades, mortality rates associated with biliary tract infections have dramatically decreased, dropping from over 50% in the 1970s to less than 10% in the 1980s.^[2]^ Despite these improvements, untreated severe infections can lead to systemic inflammation, resulting in complications such as systemic inflammatory response syndrome, sepsis, and multi-organ dysfunction, with fatality rates ranging from 11% to 27%.^[3]^

In China, the prevalence of cholecystitis ranges from 0.78% to 3.91% in adults. Additionally, women are more prone to gallstones than men, with the male-to-female ratio increasing from 1.07 to 1.69 based on data from a study conducted across 24 provinces and cities. The occurrence of gallstones also increases with age, starting at 1.1% in individuals aged 20 to 29 years and reaching 11.2% in the general population.^[4]^

Emerging studies have identified a potential link between cholecystitis and specific immune cells. Notably, Professor Valeria Orru associates cholecystitis with immune cell CD45 on CD33br HLA-DR+.^[5]^ However, investigations into the role of these immune cells in cholecystitis remain limited. Glycine, a multifunctional amino acid essential for glutathione synthesis and 1-carbon metabolism, plays a key role in regulating methylation through the folate and methionine cycles.^[6]^ Recent studies have highlighted the antioxidant and anti-inflammatory properties,^[7,8]^ suggesting its involvement in the pathogenesis of cholecystitis.

This study explored the influence of glycine on cholecystitis development by examining its interaction with CD45 on CD33br HLA-DR+ immune cells and their role in disease progression. By employing the principles of Mendelian inheritance, Mendelian randomization (MR) mitigates the effects of measurement errors, confounding variables, and reverse causality.^[9]^ Here, we investigated the causal relationship between cholecystitis and CD45 in CD33br HLA-DR+ immune cells, as well as the mediating role of glycine levels in this association.

## 2. Materials and methods

### 2.1. Study design

**Figure 1.**
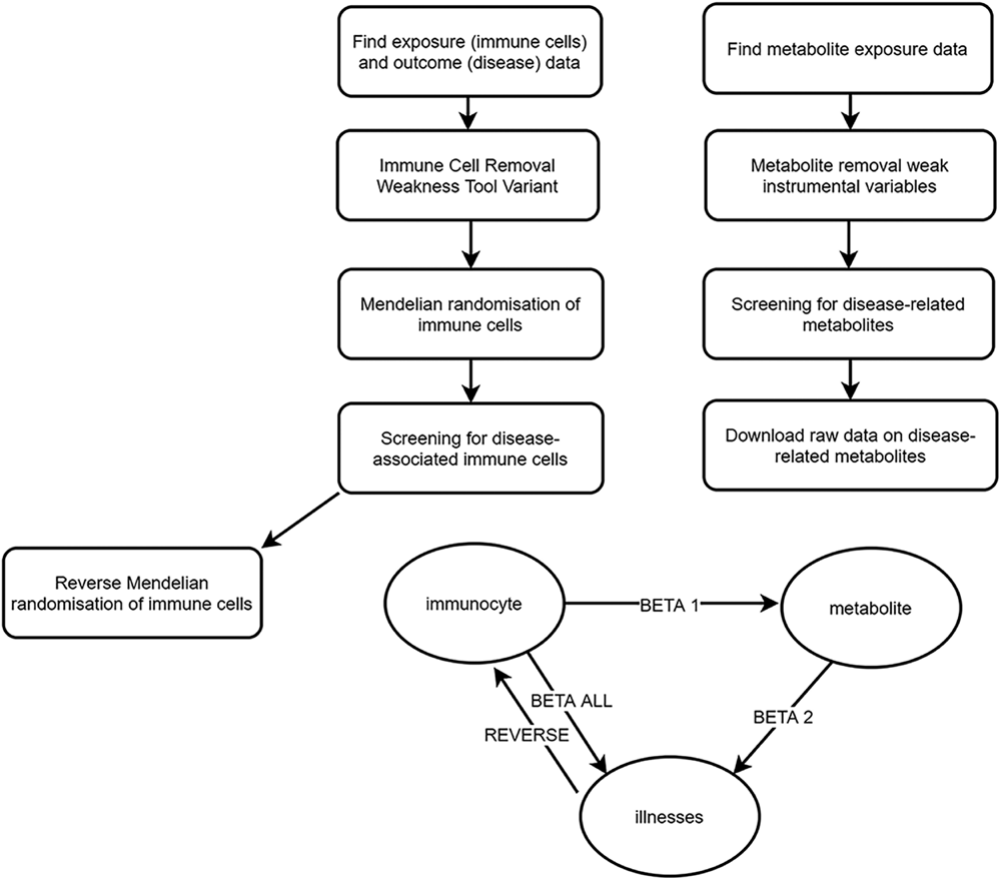
Research idea of this study: Direct effect = beta all; Indirect effect = beta1 × beta2.

This 3-phase MR study was designed to investigate causal relationships between immune cell phenotypes, metabolites, and cholecystitis risk while rigorously evaluating mediating mechanisms. The analysis adhered to the Strengthening the Reporting of Observational Studies in Epidemiology-Mendelian Randomization guidelines to ensure methodological transparency and reproducibility (Fig. 1).

### 2.2. Phase 1: exposure-outcome validation

We first identified instrumental variables (IVs) for immune cell traits and cholecystitis using genome-wide association study (GWAS) summary statistics. The primary outcome dataset included 9820 cholecystitis cases (IEU GWAS ID: ukb-b-11327), while exposure data comprised 731 immune cell phenotypes derived from the SardiNIA Project, quantifying surface protein expression levels (e.g., CD45 on CD33br HLA-DR+ cells). Genetic IVs were selected under a 2-tiered threshold: a genome-wide significance threshold (*P* < 5 × 10^−8^) was applied primarily, with a secondary threshold (*P* < 5 × 10^−5^) used when fewer than 10 IVs were available. To mitigate linkage disequilibrium (LD) bias, clumping was performed (*r*^2^ < 0.001, clumping window = 10,000 kb). Causal effects were estimated via inverse variance weighting (IVW) regression, supplemented by sensitivity analyses (MR-Egger, weighted median) to address potential pleiotropy.

### 2.3. Phase 2: mediation analysis

**Figure 2.**
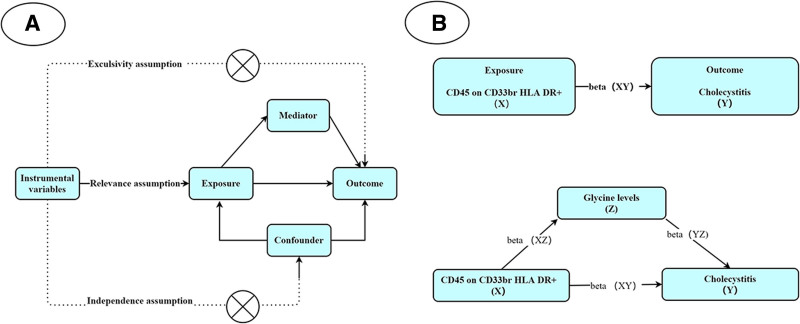
Overview of the research design. (A) Three key assumptions for MR analysis. (B) Mediation analysis. MR = Mendelian randomization.


To dissect the mechanistic pathway linking immune cells to cholecystitis, we conducted a 2-step MR analysis (Fig. 2B). Glycine, a metabolite implicated in inflammatory regulation, was selected as the mediator using metabolomics GWAS data. First, we estimated the effect of CD45 on CD33br HLA-DR+ cells on glycine levels (beta1). Second, we quantified the glycine-cholecystitis association (beta2). The total effect (beta_all) was decomposed into direct (immune cell → cholecystitis) and indirect (immune cell → glycine → cholecystitis) pathways using the product-of-coefficients method:

$$\begin{array}{l} Beta\;direct=Betaall-(beta1\times beta\;2), \end{array}$$

with mediation proportion calculated as (beta1 × beta2)/beta all.

### 2.4. Phase 3: sensitivity framework

Robustness of findings was assessed through a multilayered sensitivity framework: Heterogeneity: Cochran *Q* statistic evaluated heterogeneity across IVs. Pleiotropy: MR-PRESSO (Mendelian Randomization Pleiotropy RESidual Sum and Outlier) detected and corrected horizontal pleiotropy (*P* < .05 for global test). Stability: Leave-one-out analysis iteratively excluded individual single nucleotide polymorphisms (SNPs) to confirm result consistency.

### 2.5. Adherence to MR assumptions

All analyses complied with MR core assumptions: IVs were strongly associated with exposures (*F*-statistic > 10), IVs were independent of confounders, and effects on outcomes were exclusively mediated through exposures (Fig. 2A). Publicly available GWAS datasets were utilized to minimize population stratification, and all statistical workflows were implemented in R using two-sample Mendelian randomization (TSMR) and MR-PRESSO packages.

### 2.6. Summary of data sources

For this study, we utilized data on CD45 on CD33br HLA-DR+ immune cells, glycine levels as a mediator, and cholecystitis cases from the works of Valeria Orru, Yiheng Chen,^[10]^ and the IEU Open GWAS database. A summary of the GWAS data on cholecystitis is available at https://gwas.mrcieu.ac.uk. The dataset included 9820 cholecystitis cases and 4,614,318 participants, with the trait “cholecystitis” representing the condition.

Immune cell exposure data were obtained from Valeria Orru study, encompassing information on 731 types of Sardinian immune cells. In the initial step of MR analysis, SNPs were screened using a significance threshold of *P* < .00005, leading to the identification of an SNP associated with cholecystitis that served as an IV. LD among SNPs was then resolved using the parameters Kb = 10,000 and *r*^2^ = 0.001.

Data on glycine metabolite levels were obtained from Chen et al and included information on 1400 metabolites. SNPs significantly associated with glycine levels were filtered using the same *P* < .00005 threshold, with relevant SNPs subsequently identified as IVs for the MR analysis. Similarly, LD-related SNPs were similarly excluded using Kb = 10,000 and *r*^2^ = 0.001.

### 2.7. IV selection

To explore the causal relationship between exposure (immune cells and glycine) and outcome (cholecystitis), an MR framework was employed using SNPs as IVs.^[11]^ The selected IVs had to meet 3 critical criteria: a strong association was observed between exposure (e.g., immune cells) and the IVs; there was no direct association between the IVs and the outcome (cholecystitis); independence of IVs on confounding factors.^[12]^

A genome-wide significance threshold of *P* < 5 × 10^−8^ was used to identify SNPs associated with cholecystitis, glycine levels, and CD45 in CD33br HLA-DR+. When the number of IVs was insufficient, a relaxed threshold of *P* < 5 × 10^−5^ was applied to increase the pool of exposure-related IVs. Stringent criteria were applied to minimize bias from LD, requiring *r*^2^ < 0.001 and a distance of >10,000 kilobases.^[13]^

Horizontal pleiotropy was assessed and addressed using the MR-PRESSO method,^[14]^ which excludes outlier SNPs. Palindromic SNPs with intermediate allele frequencies were removed and *F*-statistics were calculated to evaluate the strength of the genetic instruments. SNPs with an *F*-value below 10 were considered weak and excluded from the analysis.^[15]^

### 2.8. MR and statistical analysis

Using R version 4.3.2 (R Core Group, Vienna, Austria), a TSMR software package was used to perform the analyses. Causal relationships between exposure and outcome were evaluated using IVW.^[16]^ Additional analytical methods, including MR-Egger,^[17]^ weighted median,^[18]^ simple mode, and weighted mode,^[19]^ were applied to support the robustness of the findings. Odds ratio (OR) and 95% confidence intervals (CI) were calculated to assess the effects on the risk of cholecystitis. The IVW analysis demonstrated statistical significance at a *P*-value threshold of .05, with consistent OR directions across methods (all >1 or <1) and a *P*-value > .05 for pleiotropy.

### 2.9. Reverse TSMR

To explore the reverse causal relationship between cholecystitis and CD45 in CD33br HLA-DR+, TSMR analysis was conducted with a significance threshold of *P* < 5 × 10^−8^ for IV selection. SNPs were identified. MR analysis found no statistically significant correlation between cholecystitis and CD45 on CD33br HLA-DR+ (*P* > .05). Furthermore, no evidence of heterogeneity or horizontal pleiotropy was found (Table 1). These results suggest no causal association between cholecystitis exposure and CD45 on CD33br HLA-DR+ cells (*P* > .05).

**Table 1:** Sensitivity analysis, including heterogeneity test and horizontal pleiotropy test.

Heterogeneity test							
Exposure	Outcome	Heterogeneity test (MR-Egger)			Heterogeneity test (IVW)		
		Cochrane *Q*	Q_df	Q_pval	Cochrane *Q*	Q_df	Q_pval
CD45 on CD33br HLA-DR+	Cholecystitis	17.4	14	0.234	17.7	15	0.278
Glycine levels	Cholecystitis	22.7	20	0.305	23.7	21	0.306
CD45 on CD33br HLA-DR+	Glycine levels	7.64	13	0.866	8.12	14	0.882

Horizontal pleiotropy test							
Exposure	Outcome	Horizontal pleiotropy test (MR-Egger)			Horizontal pleiotropy test (MR-PRESSO)		
		Intercept	SE	*P*-value	Global test *P*-value		
CD45 on CD33br HLA-DR+	Cholecystitis	0.00365	0.00783	.648	.392		
Glycine levels	Cholecystitis	0.00530	0.00552	.348	.397		
CD45 on CD33br HLA-DR+	Glycine levels	−0.00560	0.00804	.498	.878		

IVW = inverse variance weighting, MR = Mendelian randomization, MR-PRESSO = Mendelian Randomization Pleiotropy RESidual Sum and Outlier.

### 2.10. Mediating role of glycine levels

This study also investigated the potential mediating effect of glycine levels in the relationship between CD45 on CD33br HLA-DR+ and cholecystitis. The results indicate that glycine levels may exert a mediating causal effect, accounting for 6.87% of the total effect.

### 2.11. Sensitivity analysis

Three approaches were employed for sensitivity analysis: heterogeneity assessment, evaluation of pleiotropy, and the leave-one-out method. Heterogeneity was assessed using Cochrans *Q*-test, with a significance threshold of *P* < .05, which indicated the presence of heterogeneity. Horizontal pleiotropy was evaluated using the IVW method with random effects and heterogeneity.^[20]^ The MR-Egger intercept demonstrated statistical significance, indicating horizontal pleiotropy. The MR-PRESSO global test was used to evaluate pleiotropic effects further.^[21]^ Additionally, systematic leave-one-out analysis was conducted to assess the influence of individual SNPs on causal relationships by sequentially excluding each SNP.

### 2.12. Mediation analyses

Using a 2-step MR approach, we investigated how glycine levels may act as mediators in the causal link between CD45 on CD33br HLA-DR+ cells and cholecystitis. The total impact was separated into indirect (influenced by glycine levels) and direct (not influenced by glycine levels) impacts. We further deconstructed CD45 on CD33br HLA-DR+ on the total effect of CD45 on CD33br HLA-DR+ on cholecystitis into the direct effects of CD45 on CD33br HLA-DR+ and the indirect effects through glycine levels mediated through CD45 on CD33br HLA-DR+. The percentage of total effect of the mediator effect was determined by dividing through the division of the indirect effect by the mediator effect. Additionally, 95% CIs were computed using the delta method.

### 2.13. Data visualization

Sequential exclusion of SNPs allows the assessment of whether a single SNP significantly influences the outcome. Forest plots were used to visualize the estimated effects of genetic variation on the outcome. The relationship between CD45 on CD33br HLA-DR+, glycine levels, and cholecystitis was analyzed using the IVW method. Funnel plots were used to evaluate the publication bias by examining their symmetry.

## 3. Results

### 3.1. Causal associations between CD45 on CD33br HLA-DR+ expression and clinical outcomes

**Figure 3.**
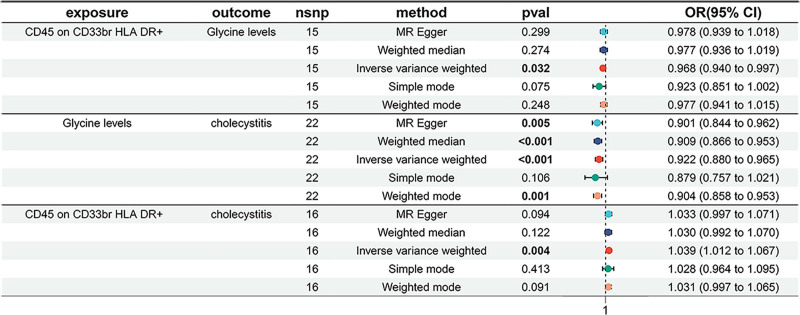
Two-sample MR analysis results. The causal relationship between glycine levels, CD45 on CD33br HLA-DR+ cells, and cholecystitis was evaluated using different MR methods. An OR value greater than 1 indicates that the exposure index will increase the risk of the result; otherwise, it will reduce the risk. CI = confidence interval, MR = Mendelian randomization, OR = odds ratio.

In Figure 3, MR analyses demonstrated a significant positive causal relationship between CD45 on CD33br HLA-DR+ cells and cholecystitis risk (IVW OR = 1.039, 95% CI = 1.012–1.067, *P* = .004). A consistent directional association was observed across MR-Egger and weighted mode methods. Additionally, CD45 on CD33br HLA-DR+ expression showed an inverse relationship with glycine levels (IVW OR = 0.968, 95% CI = 0.940–0.997, *P* = .032), suggesting potential metabolic regulation.

### 3.2. Protective effect of glycine against cholecystitis

Four complementary MR approaches (MR-Egger, weighted median, IVW, and weighted mode) uniformly identified glycine as protective against cholecystitis. The consistency across methods with narrow CIs strengthens the evidence for glycine’s causal role in cholecystitis pathogenesis.

### 3.3. Dose–response relationships and pleiotropy assessment

**Figure 4.**
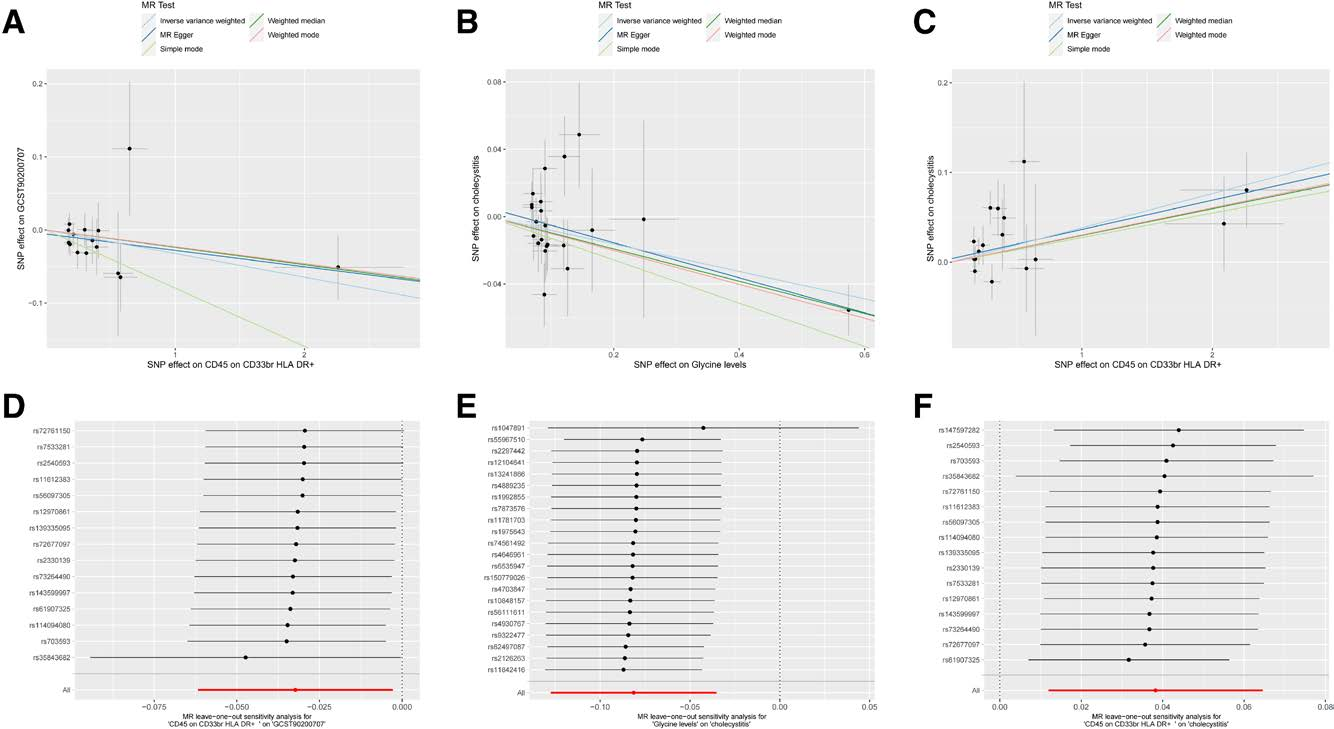
The scatter plot and “leave-one-out” results of the genetic correlation between CD45 on CD33br HLA-DR+, glycine levels, and cholecystitis were obtained using different MR analysis methods. GCS90200707 in (A) is the marker ID of the metabolite glycine level. (A and D) CD45 on CD33br HLA-DR+ cells on GCS90200707. (B and E) glycine levels on cholecystitis. (C and F) CD45 on CD33br HLA-DR+ cells on cholecystitis. MR = Mendelian randomization, SNP = single nucleotide polymorphism.

**Figure 5.**
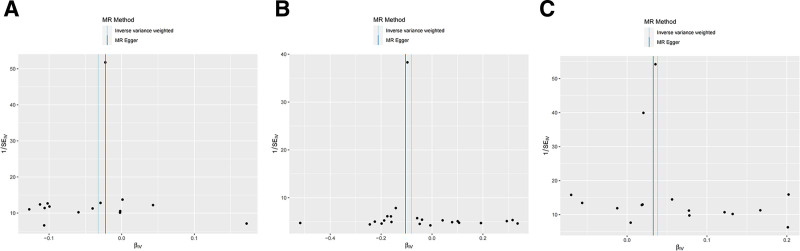
Funnel plots for the heterogeneity test. (A) CD45 on CD33br HLA-DR+ cholecystitis at glycine levels. (B) Glycine levels in patients with cholecystitis. (C) CD45 on CD33br HLA-DR+ cells in cholecystitis.

Scatter plot analyses revealed: an inverse correlation between glycine levels and CD45 on CD33br HLA-DR+ expression, and increasing cholecystitis risk with both glycine depletion and CD45 on CD33br HLA-DR+ elevation (Fig. 4A–C). Rigorous pleiotropy testing (MR-Egger intercept *P* > .05, MR-PRESSO global test *P* > .05) and funnel plot symmetry (Fig. 5) confirmed the absence of confounding horizontal pleiotropy.

### 3.4. Robustness of causal inference

Sensitivity analyses demonstrated: no heterogeneity in primary associations (Table 1, Cochran *Q*, *P* > .10), stable effect estimates in leave-one-out analysis (Fig. 4D–F), and quantifiable mediation effects (Table 2). These findings collectively validate the precision and reliability of the causal estimates.

**Table 2:** Calculation of the mediating effect.

Immune cell	Metabolite	Outcome	Mediated effect	Mediated proportion	*P*-value
CD45 on CD33br HLA-DR+	Glycine levels	Cholecystitis	0.002 (4.87e−05, 0.005)	6.87% (0.127%, 0.136)	.046

## 4. Discussion

Our findings from MR analysis suggest a causal relationship between CD45 on CD33br HLA-DR+ immune cells and cholecystitis in the European population, providing a novel perspective on the pathogenesis of the disease and identifying potential metabolic targets for treatment. The rising incidence of cholecystitis in recent years may be linked to improved living standards and irregular daily routine. After consuming greasy foods or large meals, individuals often experience right upper abdominal pain accompanied by a positive Murphy sign and, in some cases, fever, serving as a key diagnostic indicator.^[22]^

Gallbladder stones or calculous cholecystitis are found in ≥95% of cholecystitis cases. Small stones in the gallbladder can migrate into the bile duct, forming bile duct stones and potentially leading to cholangitis.^[23,24]^ If obstruction occurs at the duodenal papilla, it may result in biliary pancreatitis.^[25,26]^ Approximately 5% of patients with cholecystitis do not have gallstones, a condition known as noncalculous cholecystitis,^[27]^ with evidence suggesting immune cell involvement in its pathogenesis.^[28]^

Glycine, a nonessential amino acid with a molecular weight of 75, is an organic compound that is naturally present in the human body. It plays a role in nucleic acid synthesis and possesses anti-inflammatory and immunomodulatory properties. Glycine also contributes to bile acid formation and phosphocreatine metabolism, but its therapeutic potential remains incompletely understood.^[29,30]^ Recent studies have suggested that glycine may be involved in immune cell activation, particularly in conditions such as elevated CD45 on CD33br HLA-DR+ levels and cholecystitis. Our research revealed a correlation between increased CD45 on CD33br HLA-DR+ immune cells expression and decreased glycine levels, which collectively heightened the risk of cholecystitis. Conversely, lower CD45 on CD33br HLA-DR+ levels was associated with a reduced risk.

Using TSMR, we established a significant causal relationship between CD45 on CD33br HLA-DR+ levels and cholecystitis. As CD45 on CD33 br HLA-DR+ increased, as did the risk of cholecystitis. However, reverse TSMR results showed no direct causal link between the two, suggesting the involvement of mediating factors such as glycine, in this relationship. Despite its importance, the role of glycine in cholecystitis has largely been overlooked in academic research. Our TSMR approach demonstrated that glycine levels act as mediators in the association between CD45 on CD33 br HLA-DR+ cell and cholecystitis.

To explore the link between exposures and outcomes, we employed SNPs as IVs, leveraging the strengths of MR to minimize bias from confounding variables and reverse causality. A TSMR approach was used to evaluate linear associations, while mediation analysis was used to examine potential nonlinear relationships. This study exclusively utilized data from European populations to reduce potential bias due to population heterogeneity. Sensitivity analyses were preformed to ensure the robustness and reliability of the results.

Nevertheless, it is important to acknowledge the limitations of the present study. Most of the data were derived from European populations, necessitating further research to validate the findings across other ethnicities. Additionally, the relationship between CD45 on CD33br HLA-DR+ cells and cholecystitis is influenced by numerous confounding factors that this study could not entirely control. Future research is essential to address these limitations and to expand the generalizability of our findings.

## 5. Conclusions

In conclusion, this study, based on MR analysis, provides genetic evidence supporting a causal link between CD45 on CD33br HLA-DR+ immune cells and cholecystitis. Notably, elevated levels of CD45 on CD33br HLA-DR+ cells are associated with an increased risk of cholecystitis, with glycine levels identified as a mediating factor. These findings highlight a potential therapeutic target within the metabolic pathways that contribute to the pathogenesis of cholecystitis.

## Acknowledgments

The authors thank the participants in all the GWASs used in this manuscript as well as to the investigators who made the GWAS data public.

## Author contributions

**Conceptualization:** Zheng Zhang.

**Data curation:** Xiangrui Zeng, Jinni Yao.

**Formal analysis:** Zheng Zhang, Congyu Wang, Zhe Xu.

**Funding acquisition:** Rui Song, Jinni Yao, Huaicheng Yang.

**Investigation:** Zheng Zhang, Heqiang Liao, Congyu Wang, Huaicheng Yang.

**Methodology:** Xiangrui Zeng, Huaicheng Yang.

**Project administration:** Rui Song, Heqiang Liao, Congyu Wang, Zhe Xu.

**Resources:** Zheng Zhang.

**Software:** Jinni Yao, Heqiang Liao, Congyu Wang.

**Supervision:** Jinni Yao, Zhe Xu, Huaicheng Yang.

**Validation:** Rui Song, Heqiang Liao.

**Visualization:** Rui Song, Zhe Xu, Huaicheng Yang.

**Writing – original draft:** Xiangrui Zeng.

**Writing – review & editing:** Xiangrui Zeng, Huaicheng Yang.
